# “Health in” and “Health of” Social-Ecological Systems: A Practical Framework for the Management of Healthy and Resilient Agricultural and Natural Ecosystems

**DOI:** 10.3389/fpubh.2020.616328

**Published:** 2021-01-28

**Authors:** Michel De Garine-Wichatitsky, Aurélie Binot, John Ward, Alexandre Caron, Arthur Perrotton, Helen Ross, Hoa Tran Quoc, Hugo Valls-Fox, Iain J. Gordon, Panomsak Promburom, Rico Ancog, Richard Anthony Kock, Serge Morand, Véronique Chevalier, Will Allen, Waraphon Phimpraphai, Raphaël Duboz, Pierre Echaubard

**Affiliations:** ^1^Animals, health, Territories, Risks and Ecosystem (ASTRE), University of Montpellier, Agricultural research for Development (CIRAD), National Research Institute for Agriculture, Food and the Environment (INRAE), Montpellier, France; ^2^Agricultural research for Development (CIRAD), UMR ASTRE, Bangkok, Thailand; ^3^Faculty of Veterinary Medicine, Kasetsart University, Bangkok, Thailand; ^4^Mekong Region Futures Institute, Bangkok, Thailand; ^5^Universidad E. Mondlane, Maputo, Mozambique; ^6^Stockholm Resilience Center, Stockholm University, Stockholm, Sweden; ^7^UMR Eco&Sols, IRD, Agricultural Research for Development (CIRAD), Montpellier, France; ^8^School of Agriculture and Food Sciences, The University of Queensland, Brisbane, QLD, Australia; ^9^Agricultural Research for Development (CIRAD), Research unit Fonctionnement écologique et gestion durable des agrosystèmes bananiers et ananas (GECO), Vientiane, Laos; ^10^Agricultural Research for Development (CIRAD), Research Unit Systèmes d'élevage méditerranéens et tropicaux (SELMET), PPZS, Dakar, Sénégal; ^11^Research Unit Systèmes d'élevage méditerranéens et tropicaux (SELMET), Univ Montpellier, Agricultural Research for Development (CIRAD), National Research Institute for Agriculture, Food and the Environment (INRAE), Institut Agro, Montpellier, France; ^12^Fenner School of Environment and Society, The Australian National University, Canberra, ACT, Australia; ^13^James Hutton Institute, Aberdeen, United Kingdom; ^14^Central Queensland University, Townsville, QLD, Australia; ^15^Land and Water, Commonwealth Scientific and Industrial Research Organisation (CSIRO), Townsville, QLD, Australia; ^16^Center for Agricultural Resource System Research Chiang Mai University, Chiang Mai, Thailand; ^17^School of Environmental Science and Management, University of the Philippines Los Baños, Philippines; ^18^Department of Pathobiology and Population Sciences, Royal Veterinary College, London, United Kingdom; ^19^Centre national de la recherche scientifique (CNRS), Institut des Sciences de l'Evolution de Montpellier (ISEM), Faculty of Veterinary Technology, Kasetsart University, Bangkok, Thailand; ^20^Epidemiology Unit, Institut Pasteur du Cambodge, Phnom Penh, Cambodia; ^21^Learning for Sustainability, Christchurch, New Zealand; ^22^Sorbonne Université, IRD, UMMISCO, Bondy, France; ^23^School of Oriental and African Studies (SOAS), University of London, London, United Kingdom

**Keywords:** health, biodiversity, agriculture, social-ecological systems, resilience, co-learning

## Abstract

The past two decades have seen an accumulation of theoretical and empirical evidence for the interlinkages between human health and well-being, biodiversity and ecosystem services, and agriculture. The COVID-19 pandemic has highlighted the devastating impacts that an emerging pathogen, of animal origin, can have on human societies and economies. A number of scholars have called for the wider adoption of “*One Health* integrated approaches” to better prevent, and respond to, the threats of emerging zoonotic diseases. However, there are theoretical and practical challenges that have precluded the full development and practical implementation of this approach. Whilst integrated approaches to health are increasingly adopting a social-ecological system framework (SES), the lack of clarity in framing the key concept of resilience in health contexts remains a major barrier to its implementation by scientists and practitioners. We propose an operational framework, based on a transdisciplinary definition of Socio-Ecological System Health (SESH) that explicitly links health and ecosystem management with the resilience of SES, and the adaptive capacity of the actors and agents within SES, to prevent and cope with emerging health and environmental risks. We focus on agricultural transitions that play a critical role in disease emergence and biodiversity conservation, to illustrate the proposed participatory framework to frame and co-design SESH interventions. Finally, we highlight critical changes that are needed from researchers, policy makers and donors, in order to engage communities and other stakeholders involved in the management of their own health and that of the underpinning ecosystems.

## Introduction

The past two decades have seen an accumulation of theoretical and empirical evidence for the interlinkages between human health and well-being, biodiversity and ecosystem services, and agriculture (1, 2). The emergence of infectious diseases associated with human manipulations of animal species and their habitats can have significant impacts on human societies and economies, and on biodiversity conservation (3–5). The COVID-19 global crisis illustrated how devastating and persistent such a pandemic can be, calling for major changes of human-animal interactions: “If no changes are made, it is inevitable that zoonotic pathogens will continue to emerge and threaten global health and economies” (6). However, this is far from the first major pandemic in the history of humankind (7, 8). Major changes in attributes and intensity of agriculture, and the domestication of livestock species, had an important impact in perturbing local value chains and natural resources management, thereby amplifying the transmission rate of pathogens from animals to humans (9). The frequency and magnitude of emerging zoonotic diseases outbreaks have increased in recent decades, with a sequence of epidemics suspected to have resulted from human practices directly or indirectly impacting on wildlife ecology: Avian Influenza viruses, Nipah virus, SARS-Cov-1, MERS-CoV, and SARS-Cov-2, to name the most deadly. While there has been a proliferation of proposed approaches for improved and concerted human and animal health and environmental management, the lack of a common, coherent framework (10) and a consensus on what defines healthy social-ecological systems (SES) (11) have impeded operational implementation thus far (12, 13).

The productivity paradigm that has been dominating since the industrial revolution (14, 15) has brought human activities beyond Earth's capacity to sustain them, and many of the current public health challenges are directly linked to the degradation of ecosystems and the services they provide to humanity (16, 17). A decade ago, Rockström et al. (18) highlighted how the boundaries for a safe operating space for humanity have already been exceeded for several essential interlinked planetary systems, including climate change and rate of biodiversity loss, both linked to direct impacts on human, animal and environmental health (18). These trends have worsened, and additional key parameters are even more rapidly closing in on the safe boundaries (19). This is the case of global freshwater use (20) and the rate of land use conversion (19), which are two of the main factors associated with the emergence of human pathogens (21), and of biogeochemical flows of Nitrogen and Phosphorus. Among all the human activities that have detrimental environmental impacts, capitalistic intensive agriculture, part of a complex political ecology in which global to local dynamics of social and political power shape social-ecological change (22, 23), is a major force behind some of the most significant threats (24). This includes the conversion of natural habitats, degradation of soils and freshwater, and the contribution of greenhouse gases (25, 26). All these parameters have also been shown to impact negatively on the health of people, animals and plants. With the global human population expected to rise to between 9 and 11 billion by 2050, sustainable agriculture, food security and global health are at the forefront of the global development agenda (27, 28).

Among the diverse array of opinions and recommendations on COVID-19 crisis management, several scholars have called for a *One Health* approach (29–32) echoing earlier calls for the management of MERS coronavirus outbreak (33). The recognition of the interdependencies between the health of humans, non-human-animals and ecosystems, may seem relatively new for the general public and some decision-makers, although it has already generated a considerable amount of literature (34). Since the initial elaboration of an “ecosystem approach to human health” (35), several systemic approaches to health have been developed, including the *EcoHealth* (36) and *One Health* initiatives (37), ultimately converging (38, 39), and Planetary Health. There has been increasing acknowledgment, at least among scientists and some policy makers, that health and environmental issues must be managed holistically across multiple bio-physical, economic and social scales and across landscape, national and global levels (40–42).

*One Health* integration has been impaired by animated debates between divergent disciplines (38, 39, 43), competing schools of thought (44) and delayed convergence of relevant systemic and participatory modeling approaches (45, 46), that have constrained effective interdisciplinary and cross-sectorial collaborations (47). While efforts have been made to implement *One Health* approaches in practice (48, 49), there is still an acute need to operationalize health management based on a social-ecological system and resilience framework (12, 13) that recognizes power dimensions in the “coupling” of human and natural systems (22, 23). The multiplicity of competing “systemic holistic approaches” to health have added to the confusion (39, 50). Antoine-Moussiaux et al. (10) argue that the main barrier to inter- and trans-disciplinary solutions to improve the management of health risks and benefits lies in the lack of reflexivity and reflection by scientists about their respective operational framing, which is also acknowledged by Wilcox et al. (12), along with the ill-defined problem structuring of policy makers (51). In this paper we highlight the main theoretical and practical challenges that have precluded the full development and implementation of collaborative and participatory integrated approaches that support collective actions in health. We propose an operational framework, based on a transdisciplinary definition of Social-Ecological System Health (SESH) explicitly linking health and ecosystem management with the adaptive capacity of the actors and agents of coupled social-ecological system to prevent and cope with emerging health and environmental risks. We focus on agricultural transitions which play a critical role in both disease emergence and biodiversity conservation, and highlight critical process changes that are needed from researchers, practitioners, policy makers and donors, in order to engage communities and other stakeholders involved in the management of their health and that of the ecosystems that underpin it.

## Methods

### Social-Ecological System Health: Framing Health in Nature and Society

**Figure 1:**
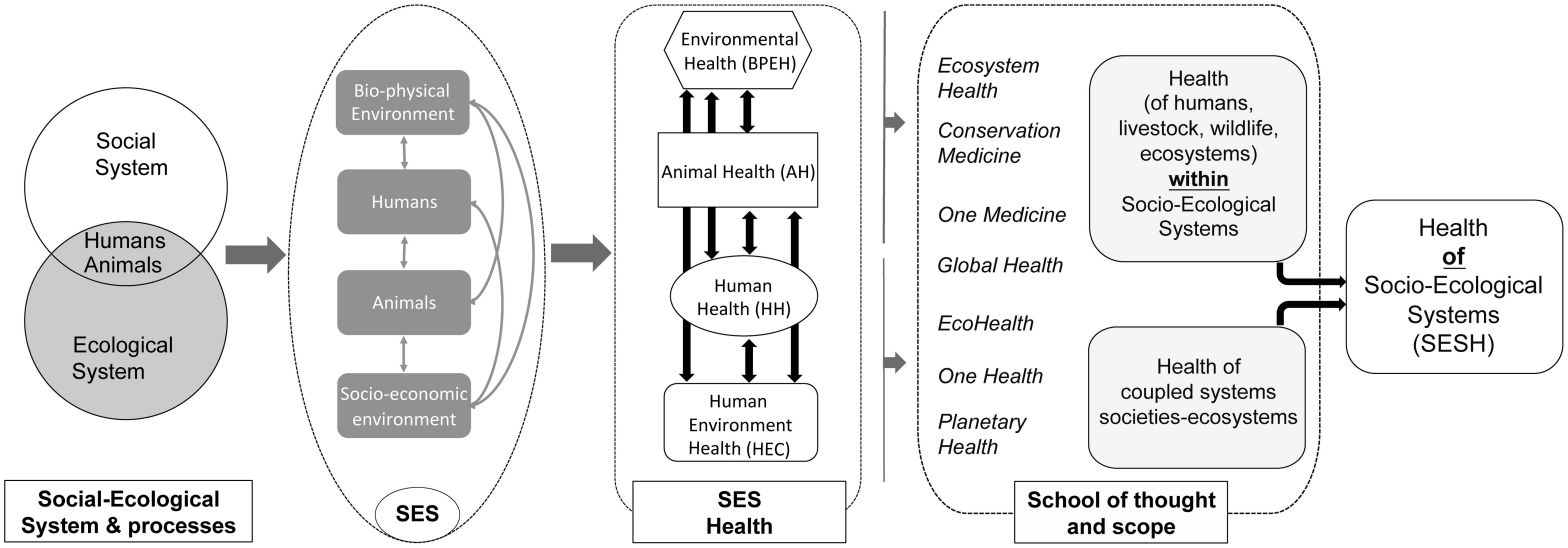
Expanding the concept of social-ecological systems to Social-Ecological System Health. Conceptual model connecting a social-ecological system template to the health of its components and the health of the whole social-ecological system (left and central parts of the graph); main integrated approaches associated with the specific health components and their interactions [definitions as in (12, 34); right part of the graph]. BPEH, bio-physical environment health; AH, Animal Health; HH, Human Health; HEC, Human Environment Health; SESH, Social-Ecological System Health.

A critical step in inter-disciplinary and cross-sectoral *One Health* collaborations lies in the way questions and issues are framed (10, 13), especially when addressing complex inter-linkages such as the Health-Biodiversity-Agriculture nexus (52). Despite repeated early calls for closer collaboration, the medical and veterinary spheres (53, 54), and the environmental (55) and social sciences (37, 56), have struggled to establish strong, long-lasting collaborations grounded on a clear shared framework (10, 57). *One Health* has been presented as an approach to address health threats at the “human-animal-environment interface”, also referred to as “human-animal-ecosystem interface” (34, 58), with both formulations used interchangeably by the same operators, including the tripartite coalition of UN agencies spearheading the concept (59, 60). Beyond the semantic debate, these ambiguities illustrate the confusion as to the framing of the proposed systemic approach, which has been a major factor contributing to the misunderstanding between disciplines (56), and a barrier for inter- and trans-disciplinary solutions to improve inter-sectoral management of health risks and benefits (10). Clearly it is of paramount importance to define the boundaries and the components of the complex system through which the approach analyses health and environmental issues, as it may refer to very different definitions of health and contrasted views about human-nature relationships (i.e., are humans, and non-human animals, part of ecosystems or outsiders?), which are supported by distinct disciplines and management sectors. Defining and comparing all the various holistic approaches to health that have been proposed in recent decades is beyond the scope of this paper, and we refer to recent review papers for an exhaustive list and more details regarding each approach (12, 34, 50). As illustrated in Figure 1, approaches to health and environmental management have progressively converged (61), and two main frameworks should be distinguished, based on the spatio-temporal boundaries of the systems and the health outcomes considered by each approach:

1) Health(s) ***within*** Social-Ecological contexts: Initially presented as an analogy (health of organisms ~ health of human, or animal populations, or other components of the ecosystem), health issues have been progressively included within increasingly complex social-ecological contexts, and at larger levels: Human-Animal-Wildlife-Ecosystems-Biosphere (62). The focus of the management or research activities remains on the health of the “nested” object (human, or domestic animal, or wildlife etc.), situated in its social and ecological contexts. Different approaches have been successively defined depending on which health components of the system they focused on, and the associated disciplines (34): *One Medicine* focusing on Human-Animal interactions*, Ecosystem Health* promoting linkages between ecology and medicine, *Conservation Medicine* focusing on biodiversity conservation and wildlife health, and *Global health* placing a priority on improving health and equity for all people worldwide (63).

2) Health ***of*** Social-Ecological Systems: complex human-environment systems are best defined as coupled social-ecological systems (64, 65). The health of these complex adaptive systems has been related to the concept of resilience [SESR; (12)], as proposed seminally by Holling (66) for natural ecosystems and further adapted to social-ecological systems (12, 67). The integrated approaches under this group are bound to adopt a more holistic perspective, accounting for influences across wider temporal and spatial scales, and a wider range of stakeholders, for which transdisciplinary is essential (68). The integrated approaches under this group include *EcoHealth*, defined as systems approaches to promote the health of people, animals, and ecosystems in the context of social and ecological interactions (69), *Planetary Health*, defined as the health of human civilizations and the natural systems on which they depend (16, 70), and some of the latest developments of “One World One Health TM”/*One Health* (34). *One Health* promotes interdisciplinary collaborations to optimize the health of people, animals and the environment, which falls under “Health *within* SES” category. However, *One Health*, embedded within the concept of *EcoHealth* thinking, was further extended to complex human-environment systems (71), ultimately addressing “Health ***of*** SES” as well as “Health ***within***”. The holistic understandings of some Indigenous societies, in which human and ecosystem health are regarded as closely interdependent, are also consistent with this perspective (72, 73).

### A Transdisciplinary Context-Dependent Definition of SESH

Social-Ecological System Health is a comprehensive, multi-scale, and dynamic measure of the state/health of a functional social-ecological system, capable of delivering health and well-being resulting from the state/health status of its main components (e.g., human health, animal health, environmental health, and socio-economic health), and from the interactions among these individual health components. As suggested by Wilcox et al. (12) the resilience of such systems [SESR as defined in Social-Ecological Systems theory; (66)] is an essential property associated with their adaptive capacities. Health is a central criterion for the sustainability of social-ecological systems (36), and SESR is thus closely dependent on SESH.

**Figure 2:**
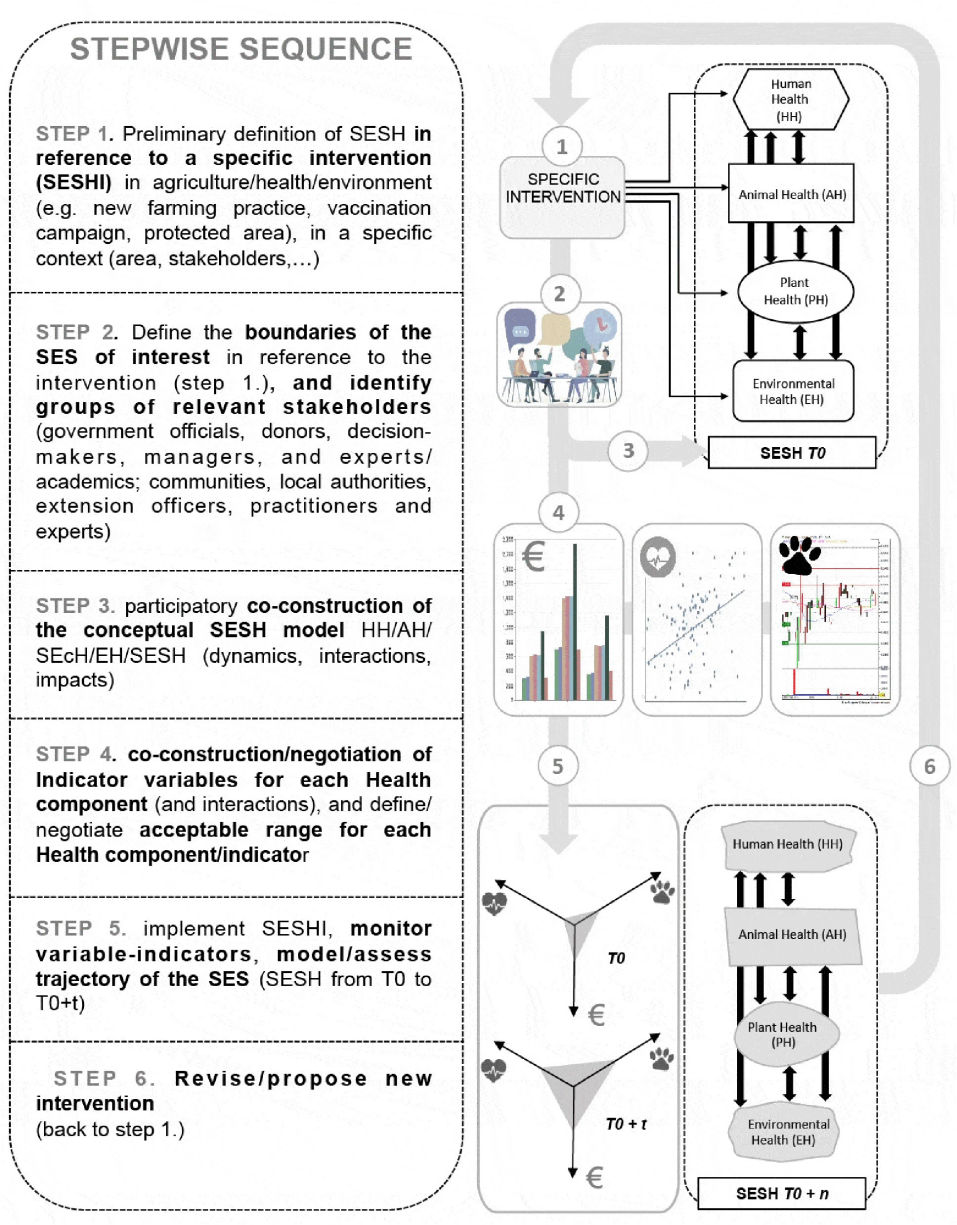
SESH operational stepwise approach.

Figure 1 illustrates how the health/state of the various components of the SES are interdependent and contribute to the health/state (i.e., resilience and its related attributes) of the whole system, including humans (and their institutions and governance systems, cultures, economic systems and power relations and influence) as an integral part of the ecosystem. As the proposed SESH concept aims to provide a catalyst for interactions between those investigating, those generating, and those responding to interlinked health and environmental issues, viewed from biomedical, ecological, socio-cultural and economic perspectives (43), the SESH operational framework explicitly includes the following components easily identifiable by the operators:

- Health of Humans (HH), Animals (AH, including domestic and non-domestic animals) as components of the health status of social-ecological systems (37), which are the focus of public and veterinary health interventions, including the prevention and control of zoonotic and vector-borne diseases and other biological threats (74). Plant Health (PH) may also be highlighted in contexts where crop production and protection are prominent (see Figure 2 and Box 2). Alternatively, plant health may refer to plant species diversity, in which case crop plants will be included in the health of the environment component (BPEH, see hereafter) associated with all plant species, often together with animal biodiversity/wildlife.

- Health of the Environment: this includes the bio-physical Environment (BPEH), which relates to actions aiming at preserving biodiversity and ecosystem integrity (75), and at maintaining environmental health above the “critical natural capital” necessary to provide essential services for the health and well-being of communities (62, 76), which are typically the focus of interventions promoting biodiversity conservation and community based natural resource management; Health of the human environment (HEH), corresponding to the components of the social, cultural and economic environments, including the institutions and legal setups, that contribute to health and well-being of communities (77) and are key components of SESH, and maybe aggregated with SESH, or singled out as Health component contributing to SESH. The Health of the Human Environment may include collective resources such as social capital (78) and solidarity (79), efficient governance systems (80), and all social determinants of health considered as public goods or within a commons health approach (81).

- Social-Ecological System Health (SESH) is directly linked to the capacity of the system to sustainably deliver health to the different constitutive components, which links to the definition of the resilience of a SES, and has been typically targeted by sustainable development and resilience building projects (12, 82).

Since the early conceptual developments of ecosystem health in the early 1990's, ecologists and environmental scientists have questioned the appropriateness of the *Ecosystem Health* concept, overwhelmingly rejecting the idea that it can be measured as an objective, quantifiable property of an ecosystem (83), and questioning the superorganism paradigm of ecosystems that assumes an equilibrium in reference to a desirable and stable state (75). In contrast, the theory of social-ecological systems has robustly defined the concept of resilience, which does not assume the existence of equilibrium states and “measures the persistence of systems and their ability to absorb change and disturbance and still maintain the same relationships between populations or state variables” (66). This approach acknowledges the possibility of the existence of multiple stable states, and resilience is related to how actors navigate systems changes across these states (67). We have also seen, in recent years, more calls for a “personalized ecology” (84), especially in line with human–nature interactions at the level of individual people, driven by concerns around “unhealthy” human–nature interactions, which have undoubtedly further increased worldwide after the COVID-19 pandemic. There are several examples that illustrate how health and well-being may be framed from the perspectives of place-based communities (85, 86). Further, elaborations of resilience thinking have pointed out the importance of power relationships (and social diversity) in creating the varying and changing social-ecological conditions and processes which frame ecosystem health (87). In essence, the characterization of SES, in which humans are part of nature, may not be independent from anthropogenic views (88). Rather, the SESH approach suggests that the state of the local SES can be defined in a context-dependent way, which may nevertheless be used as a robust reference point by a group of stakeholders in order to navigate their health and its linkages within their specific SES (89). Health, and illness, are social constructions, deeply ingrained in the culture and history of the social groups which define them (90). Similarly, SESH should be viewed as a transdisciplinary context-dependent participatory exercise, a place-based process during which the framework, its constitutive components and how they interact, are co-designed with the local stakeholders (i.e., designed collaboratively by adding/removing/modifying components and interactions in order to ensure that they match the local context, knowledge and understanding of the issues at stake). The proposed SESH framework should be flexible and negotiable according to the context and the objectives of the intervention, and should not appear as a top-down imposed view. However, it is important that the initial framework proposed for the co-design team does explicitly mention the main health components, usually targeted by each sectorial operator (human and animal health, plant and crop health, biophysical environment, human environment), and allows for the clarification between “health in” vs. “health of” ecosystems as illustrated in Figure 1.

### SESH Operational Framework

Each SESH intervention should be negotiated with key stakeholders as a transformational sustainability intervention (91) addressing a specific problem that they have identified as affecting locally their health or their environment. A shared conceptual framework is essential for such transdisciplinary initiatives open to subsequent revision, adaptation and adoption by stakeholders (92), especially for health related issues (10). We propose an operational framework divided into successive steps organized in an iterative action-research process (12, 48, 93), to co-design a context-dependent SESH conceptual model, and co-design, implement, monitor and evaluate a practical field intervention (Figure 2). We illustrate the first steps of the proposed SESH transdisciplinary process with examples of projects workshops aiming at accompanying agro-ecological transitions in agroecosystems (i.e., social-ecological systems where human manipulations alter natural ecosystems).

Firstly, the process must be acknowledged as necessary/desirable by a majority of people in the local communities in order to address a problem associated with their lived context. This should be followed by the initial identification of some particular “intervention” aiming at modifying one or several health components (e.g., improved crop health resulting from an innovation in agriculture practice, increased biodiversity following the protection of natural habitats, human health benefits of alternative livestock disease control, etc.). This step sets the reference which will begin to define the dynamics of the SES, and upon which further contextualization and integration will be built (Figure 2/Step 1).

The next steps (Figure 2/Steps 2–4) are similar to those suggested for the definition of participatory sustainability indicators (94), adopting a participatory modeling approach to define the boundaries of the SES (e.g., village, river catchment, sub-district…), involving the relevant groups and legitimate actors, co-designing the conceptual models representing the linkages between SESH and its components parts (through iterative negotiations), as well as predicting/evaluating the impacts of the intervention. Step 4 plays a key role in the participatory process as it consists of the negotiation of indicator variables that will be used to monitor the change in the SES, including developing a consensus regarding the acceptable range of each indicator variable. Such indicator variables may include quantitative variables (e.g., levels of antibiotics or pesticides residues in the aquifer, financial benefits of organic farmers, number of birds…), and qualitative variables (e.g., self-assessed individual health and well-being, organization of market-chains, innovations in agroecological practices…), identified by the stakeholders as relevant to reflect the trajectory of their SES. This key step involves co-learning and negotiation among stakeholders, including donors, managers and decision makers for performance indicators, and communities, practitioners and local authorities for monitoring of the intervention (Figure 2/Step 4). The participants are invited to share and blend local and scientific knowledge in order to co-design variables indicators of key health/conservation/resilience and evaluate the costs and logistics of the associated research and training programs. This step also clarifies roles and responsibilities regarding the measurements of the indicators and how they will be used, and by whom, thereby building ownership of the process beyond the trans-disciplinary cognitive exercise. This also allows for the identification of necessary innovations, social and otherwise, which will contribute to improve the health of individual components, and the adaptive capacity of, the SES of interest.

The last steps (Figure 2/Steps 5 and 6) are dedicated to the implementation and monitoring of the intervention, with the participatory assessment of the trajectory of the SES, in order to revise the intervention and the conceptual SESH model, back to the initial step in an adaptive management iterative loop (89).

### SESH Framework in Practice

**Figure 3:**
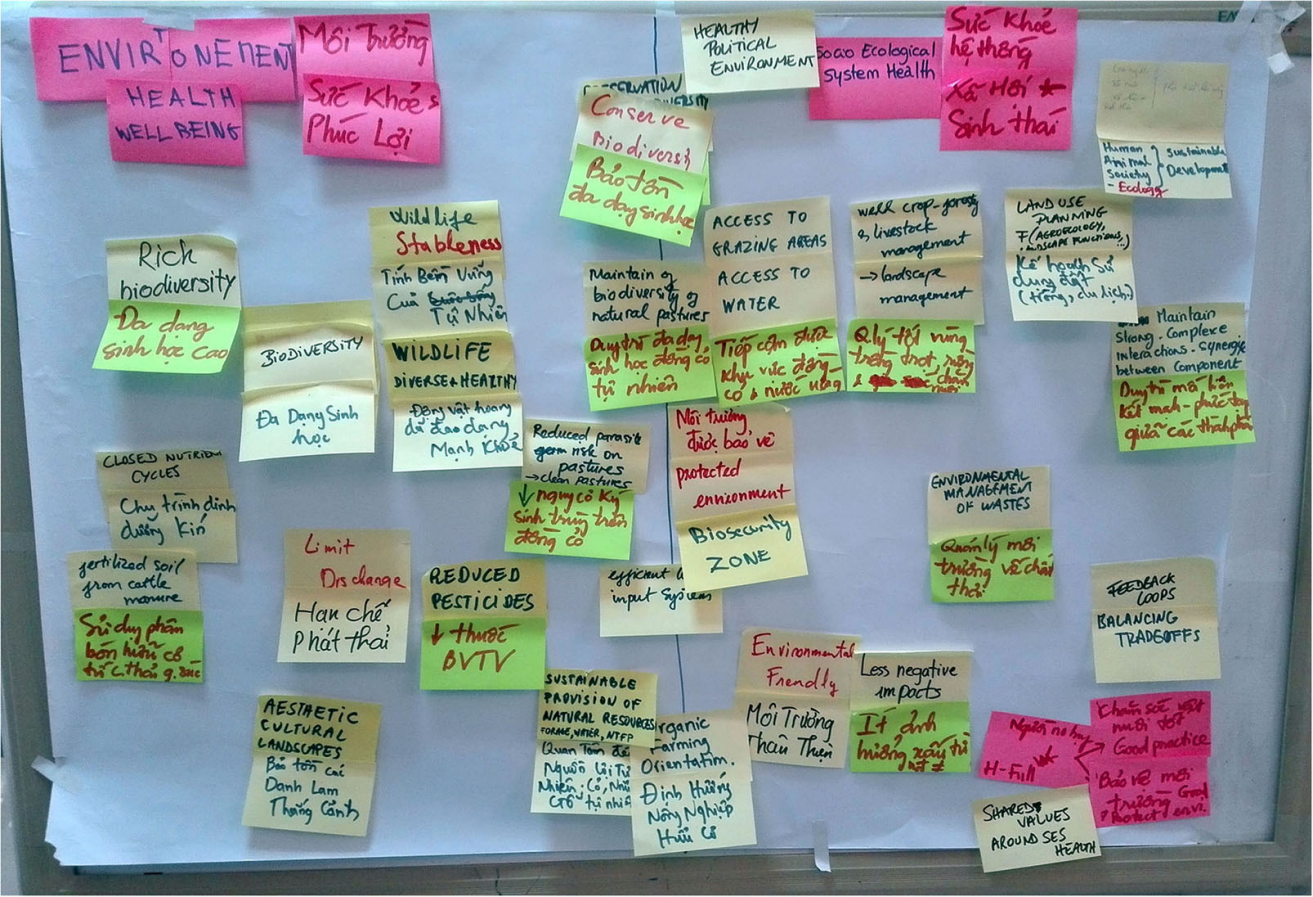
Key words and concepts suggested by participants for the collective definition of a context-based SESH of livestock-based systems (Hanoi, Vietnam, December 2019). The picture shows two the four boards produced (Environment Health, Socio-Ecological System Health) to co-design the components of the system (see Figure 2, steps 1–3). All key words and concepts were written in English and Vietnamese, and all discussions were expressed in any of these two languages and simultaneously translated.

**Figure 4:**
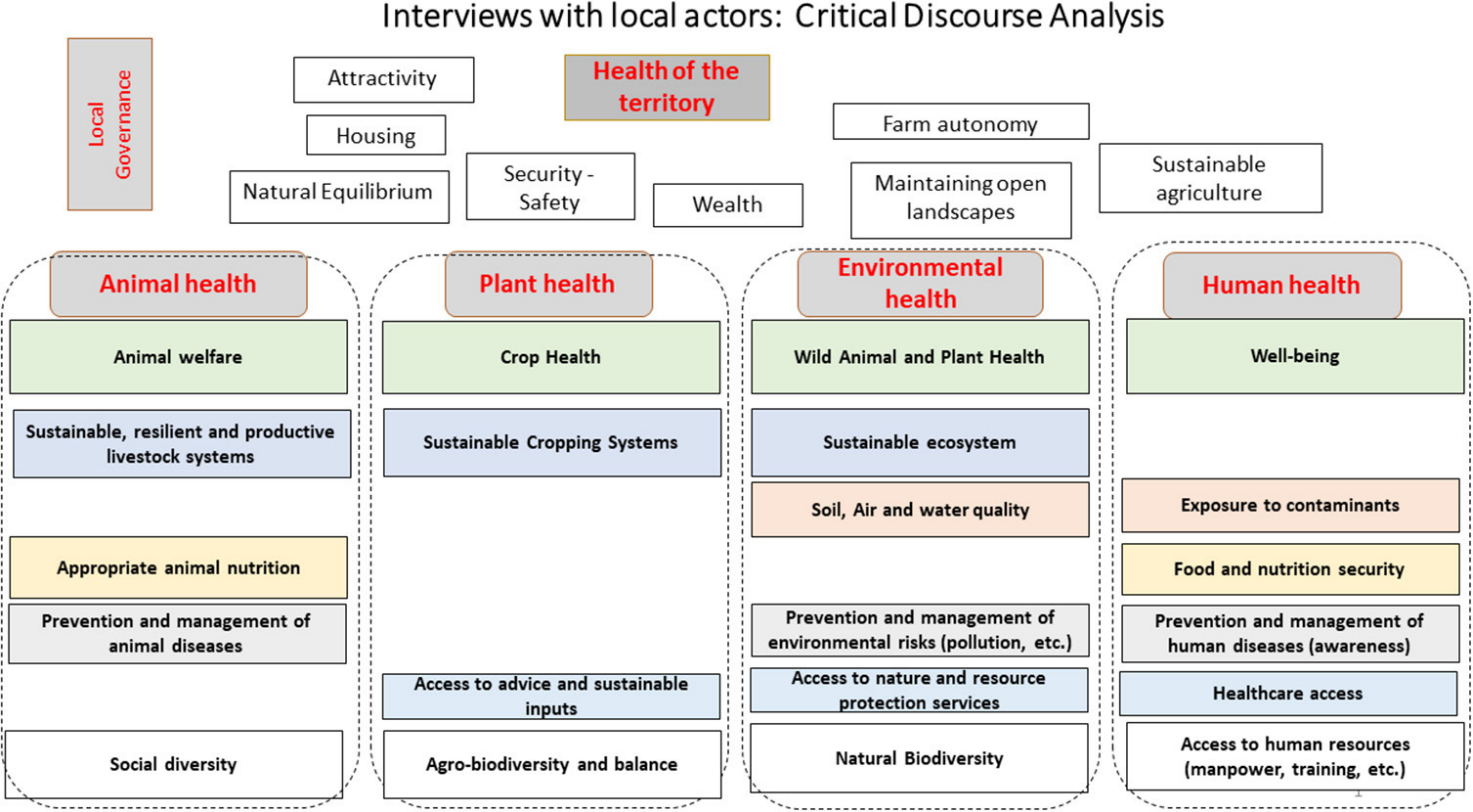
Attributes of the Health of the Territory, following a critical analysis of local stakeholders' discourses using a framework derived from SESH framework (Figure 1) to account for the specific context of agroecological transitions in Southern France.

The proposed SESH framework has not yet been used to support the full cycle of a project co-design, implementation and monitoring, as described in Figure 2. However, we report hereafter on two practical examples for which the framework has proved useful to initiate the first phases of the process (steps 1 to 3; Figure 2) in the context of agricultural transitions. We present a summary of these applications with transdisciplinary groups of researchers, agriculture extension officers and farmers in Asia (Box 1, Figure 3) and in Europe (Box 2, Figure 4).

Box 1 **Using SESH as a heuristic for a transdisciplinary vision of agro-ecological systems in transitions: livestock mobility in highlands landscapes of South-East Asia.**

**Context of application and stakes: overview of livestock-related challenges in South-East Asia**

Livestock management exemplifies the notion of a social-ecological system under transition. As a production system, livestock (i) plays an essential role in securing the livelihoods of millions of small-scale farmers; (ii) often contributes to the social identity of the place; (iii) shapes the natural environment through grazing and mobility patterns; (iv) is an interface between humans and wildlife; and, (v) is impacted by multiple social-ecological feedbacks. In South-East Asia, as in most regions across the world, livestock producers are challenged by social, economic and ecological dynamics such as competing claims for space and feeding resources, and the need to integrate crop- and livestock-systems, market fluctuations, and issues around the traceability of animals and animal products, zoonotic diseases, biodiversity loss, or climate change. These seem to call for an agro-ecological transition of the sector. Rethinking and transforming the management of livestock movements could be key to addressing some of the challenges faced by small-holder, extensive livestock production systems in SE Asia.

**A transdisciplinary group to address complex agro-ecological systems dynamics**

The issues associated with livestock mobility are particularly acute in the mountainous regions of northern SE-Asia, where this work was situated. To explore these challenges and potential solutions, we invited 20 participants from different backgrounds and expertise to participate in 3 days' workshop in Hanoi in December 2019, including: 2 livestock farmers; 1 local government services (DARD) representative from a province in the Northern highlands of Vietnam; 7 Vietnamese researchers (NIAS and TUAF); 12 regional and international researchers (KU, CIRAD, CIAT, ILRI); and, a Vietnamese-English translator.

**Objectives: Co-designing action-research activities based on a shared SESH framework**

The objective of the workshop was to produce a concept-note for a regional action-research grant application. This was done through collective discussions aiming at negotiating a shared context-based definition of SESH, which was then used to identify gaps in our understanding of livestock mobility management and related social-ecological challenges and stakes, and ultimately identify a first group of action-research questions and methods to address these.

**Implementation and outputs of the SESH process**

We mobilized the SESH framework as a heuristic to frame systemic thinking and collective discussions within this heterogeneous group. Using several facilitation methods (i.e., sticky notes, conceptual mapping, theory of change), we explored participants' visions of animal, plant, human and environmental health, and identified basic indicators for each. This stage allowed participants to enrich the shared definitions of health and well-being as well as collectively highlighting the interlinkages between SES components. We then proceeded to collectively producing a conceptual model of the SESH of livestock-based systems in the area of interest, and agreed upon the general ambitions of the upcoming project: “*Improve sustainable health and well-being of small scale livestock farmers, animals and the environment by (i) co-developing and promoting access by women and youth to innovative technologies and approaches, and, (ii) promoting healthy interactions between the components of the social-ecological system to improve the knowledge and management of livestock mobility within landscapes of SE Asia.”* Finally, we identified 4 clusters of specific objectives and related activities to achieve our ambitions: conducting a baseline survey and identifying target population for the project interventions, developing adapted tracking devices for livestock, co-designing innovative livestock management practices with a pilot group, and scaling up/scaling out these practices.

**Conclusion: the added value of using SESH-operational framework**

Challenges caused and faced by livestock production in SE Asia cover a wide range of domains and in many regards, they are wicked problems which call for innovative approaches. As a heuristic, SESH constituted a relevant frame within which all participants could think, share, discuss and collectively apprehend complex social-ecological dynamics. Throughout the 3 days' workshop, SESH proved to be an effective bridge between the disciplines and domains of knowledge represented: local knowledge and expert knowledge, veterinary sciences, ecology, epidemiology, agronomy, electronic engineering and resilience science. As an operational framework, it allowed the group to produce a concept note articulating heterogeneous visions and ambitions, and identifying practical needs and research frontiers, which corresponds to steps 1 to 3 of SESH framework (Figure 2).

Box 2 **Using SESH as a heuristic for a transdisciplinary vision of agro-ecological systems in transitions: livestock parasite control in biodiverse landscapes of Southern France.**

**Context of application and stakes: challenges of controlling ticks and tick-borne diseases at the socioecological system level in South of France (Millau)**

On the periphery of Montpellier (South of France), agricultural intensification, climate change, and various forms of land use planning, have major impacts on the health of humans, animals and the ecosystems. The management of parasite infestations on livestock farming, and in particular the risks associated with ticks and tick-borne diseases, is challenging because this problem requires consultation among stakeholders who are not used to cooperating (no dedicated institutional structure), each with a different vision of the key issues at the scale of their territory. This work focused on the area of the “Grands Causses” Regional Park, a socio-ecological system rich in biodiversity, and with a diversity of landscapes.

This region is home to numerous activities that are regulated within the framework of a charter for sustainable tourism. It hosts social groups with very varied interests, including sheep breeders who supply products to the prestigious Roquefort cheese industry. Ticks represent risks in terms of loss of sheep production performance, in terms of the risk of chemical contamination of the environment (via acaricide medication), and in relation with potential transmission of zoonotic diseases to humans (Lyme disease and Crimean-Congo haemorrhagic fever).

**A transdisciplinary process to address complex systems dynamics**

In February and March 2020, we proposed to local actors a modified SESH conceptual framework to address these risks. The SESH outlined a transdisciplinary approach aiming, in the long term, to accompany the co-construction of management principles shared by researchers, private actors, local institutions, and citizen groups. The next step in our approach aims to bring out health indicators of socio-ecological systems that make sense locally, and can guide collective actions to control and monitor ticks and tickborne diseases, and meet the needs of local actors in a context where there was no official institutional structure in place to deal with these risks.

**Objectives: define a shared framework to address local Social-Ecological System Health and identify local needs and knowledge gaps**

The project focused on launching a process for ticks and tickborne diseases with local stakeholders (medical doctors, veterinarians, breeders, technicians, biodiversity management associations, national park manager) by combining a phase of individual interviews involving the actors of the territory, and a phase of exchanges with these actors, to lay the foundations of a co-construction process based on a common representation of the health issues of the territory, which will make it possible to collectively negotiate the integrated management of the risks associated with ticks. In particular, we questioned the current methods of tick management and their consequences, the actions of surveillance, control, and prevention, the actors involved, and the vision that the local actors have of the stakes associated with a “*One Health*” type approach. Based on the analysis of the discourse of the local actors, we have updated the representations they have of the attributes of human, animal, environmental, plant and territorial health (Figure 2). This analysis was presented and discussed during a workshop where all the people interviewed were invited. This allowed us to discuss perspectives for managing the risks associated with ticks that [1] make sense at the level of this territory, [2] meet the needs of local actors, and [3] would improve the overall health of the socio-ecological system.

**Implementation and outputs of the SESH process**

The conceptual framework that guided our analysis of the discourse of local stakeholders in Millau had emerged from discussions among international researchers during a workshop (“Santé-Territoire,” Novembre 2019). The original SESH framework (Figure 1) was mobilized as a heuristic to frame systemic thinking and collective discussions. Using several facilitation methods (i.e., sticky notes, conceptual mapping, theory of change), we explored participants' visions of human health, animal health, and environmental health. Plant health was added as a component to echo the importance of crop production and agronomy in the context of agro-ecological transitions. Each health component was identified by basic cross-cutting attributes, as well as emerging attributes at the level of the territory, linked to the agroecological transition process. Then, we used this modified conceptual framework to classify the expression of ideas from local actors, and for the elicitation of their needs and priorities (Figure 4).

**Conclusion: the added value of using the SESH-operational framework**

The complex challenges identified for an integrated management of ticks and tick-borne diseases at local level called for a shared conceptual framework to elicit local stakeholder views and needs. This first step in the design of such a co-conception approach was enabled by the flexibility of the SESH, the framework being proposed as a tool to be modified and redesigned in order to fit local context's specificities and actors views and priorities. In the Millau experiment, SESH framework was first mobilized as a heuristic by researchers in order to conceptually address health at territory level and to integrate human, plant and animal health's attributes within the agroecological transition framework (Santé-Territoire). Then, we used this modified framework to elicit local stakeholders' views and needs regarding ticks and tick-borne diseases management, and to explore the desirable changes in practices, knowledge and interactions needed at local level.

### Participatory Methods for SESH Process

The practical implementation of the proposed SESH framework may draw from several tools and methods developed for similar participatory processes (45). For instance, the Companion Modeling approach (95) can be adapted to define the boundaries of the SES, to map the stakeholders' interactions and the resources they mobilize, and to co-design the conceptual SESH model and the interactions and dynamics of the SESH components in reference to the intervention (Figure 2/Steps 2–4; Box 1). Companion Modeling involves the different stakeholders of a given SES, together with decision makers and researchers in identifying the problems they face in the context of their SES, co-develop a model (interaction diagrams, maps, etc.) of the dynamics and processes specific to their SES, and simulate the expected consequences of their actions. For example, it was used for health and environment management at the scale of municipalities in Thailand (96), and with villagers in Cambodia to produce transdisciplinary epidemiological models implemented in the form of a role playing game about zoonotic disease transmission (97). The approach allowed stakeholders at local village to explore the value of cooperation between the sectors concerned (e.g., environment, agriculture and public health) and actively revise the proposed health and environment interventions. The example presented in Box 1 illustrates how a Companion Modeling approach allowed the co-design of a SESH conceptual model in relation with the intensification of livestock production in Northern Vietnam, including the definition of the system's boundaries, health components of SESH and their interactions.

Several related outcomes- and learning-based methodologies can be used to support the SESH framework intervention, and the supporting participatory modeling approach described above. For example, a Theory of Change approach (98) can provide a framework that enables stakeholders and decision makers, from all levels (local to transnational), to exchange views and visions of the future and identify the range of resources, activities, intended outcomes, and underlying causal assumptions underpinning wider program success. Allen et al. (99) illustrated how the use of an outcomes-based approach in conjunction with decision support revealed the underlying causal assumptions underpinning wider program success with a diverse group of stakeholders in Southern Asutralia including farmers, researchers, conservation managers. The use of a Theory of Change associated with a SESH logic model made the causal relationships among the health components (within/of) of agro-ecosystems more visible, and proved very useful to indicate different outcomes for the groups of stakeholders involved in the workshops addressing agricultural transitions (Boxes 1, 2).

A key aspect of the SESH process that we have not yet implemented in practice, will be the negotiation and implementation of the monitoring and evaluation system (steps 4–6, Figure 2). The negotiation of SESH indicators will be an output of both the Companion Modeling and Theory of Change processes initiated, including a consensus regarding the acceptable (“healthy”) ranges of values within which these indicators may fluctuate in response to the SESH intervention. Outcomes mapping and harvesting is a related methodology that can help increase the visibility of the boundaries, gaps, and ties that characterize social networks across the continuum of health care systems (100). The adoption of a system viability framework may allow participants to characterize a range of strategies for maintaining the long-term survival of their particular system of interest, as demonstrated in response to environmental challenges in South America (101). We suggest that this approach could be adapted to model the “negotiated viability domain” of SESH, as a measure of the co-viability of Social and Ecological Systems (102). Flexible budgets that support an adaptive management approach are also needed in order to make the operational SESH framework possible in complex environmental and social settings. It is important to have linked performance management and evaluation approaches that enable the different elements in such complex interventions to be constantly reviewed and adapted.

Co-learning among the participants is a crucial aspect for the success of SESH, and requires specific monitoring throughout the process, especially to assess whether co-learning has occurred during co-design of the model and the indicators (Figure 2/Steps 3–4), and before revising the conceptual model and revising interventions through learning loops (89) (Figure 2/Step 6). The active and systematic facilitation and measurement of learning implies that SESH projects must explicitly aim to reveal the *ex-ante* knowledge and belief orientations of decision makers, and the factors likely to either influence the fate of new knowledge and beliefs, or mobilize new knowledge configurations *ex-post* participation in the SESH process. elaborated monitoring and evaluation methods, such as those developed by Smajgl and Ward (103, 104) and applied at national and supra-national levels with decision makes of the Greater Mekong Subregion (103, 104), may be modified to the requirements of social and ecological systems health, geared toward learning throughout each step of the SESH process (Figure 2). In practice, the measurable learning exercise requires: (i) explicit articulation of stakeholders' visions of a desirable, plausible future; (ii) measurement and recording of extant causal beliefs; (iii) controlled introduction of new knowledge; and, (iv) measurement and recording of changes to causal beliefs, value orientations and attitudes throughout a structured set of facilitated discussions as a measure of learning.

## Discussion

The COVID-19 pandemic has dramatically highlighted to decision makers, managers and the general public worldwide the crucial need to understand, and adaptively manage, the complex inter-linkages between health and biodiversity, and the human and bio-physical environments. Focusing on health, both as an essential desirable state of social-ecological systems, and an expected outcome of their sustainable functioning, is a powerful way to frame sustainable development interventions. This focus on health as a strong and consensual leverage point for collective actions toward sustainable development (105) is likely to promote reconciliation of the gap between sectoral interventions in ecosystem management, biodiversity conservation and public and veterinary health (12, 47). We concur with earlier suggestions that the social-ecological system theory, and the associated concept of resilience, offer an appropriate theoretical background (12, 45, 106). However, using the concept of resilience to operationalize holistic approaches for integrated health and environmental management interventions requires clarification about the framing of issues of concern and active engagement with stakeholders at relevant levels (13).

Resilience means different things for different groups of scholars and practitioners, and it is, unfortunately, seldom clearly defined and measured, even among the members of the “resilience thinking” schools of thought (107). In health, Morand and Lajaunie (102) showed that resilience has several different meanings, for instance in psychology, sociology of health, health care or public health systems. For projects that focus only on the resilience of human and ecological communities, the implementation in practice is often less than optimal because of the absence of a common lexicon and clearly framed objectives agreed to by resilience scholars, practitioners, local communities and stakeholders (108). For integrated holistic health and environment management projects, it is of paramount importance to frame the issues related to public, veterinary and environmental health, and that these are clearly identified and articulated with reference to the resilience of coupled social-ecological systems.

The proposed operational Social-Ecological System Health (SESH) framework emphasizes the opportunity for inter-disciplinary and multi-sectoral project management teams to negotiate interventions with communities and stakeholders at an early stage through a co-designed conceptual model. A SESH participatory process allows the clarification and joint definition of the boundaries of the socio-ecosystem, and the interlinkages between the health components and attendant resilience (“Health within” vs. “Health of SES”). The proposed co-design process, which leads to the development of a common language and framing of the health and environment issues, is likely to transcend the barriers for inter- and trans-disciplinary collaboration that currently constrain collaborative inter-sectoral solutions (10, 47). In addition, and most importantly, health is a social construct (102), deeply rooted in the culture, history and norms shared within social groups and shaped by their ecosystems. The definition of healthy ecosystems is, therefore, necessarily a place-based process, likely to emerge from a transdisciplinary definition with disciplinary experts (medical doctors, veterinarians, ecologists, epidemiologists, social scientists), decision makers, local communities and stakeholders. Because such a participatory definition accounts for and understands local human, environmental, and spiritual aspects that are often overlooked in standard health assessments (86), it is likely to lead to a more consensual definition of healthy ecosystems (11), while empowering the participants to take part in the management of their health and environment.

Conventional equilibrium approaches to managing human, economic, and natural resources are prone to failure because they do not capture the dynamic interactions between humans and the constantly changing contextual environment. Health and environmental issues are often embedded in complex cross-scale and cross-sectorial interactions, and more often than not can be considered as “wicked” or “messy” issues, characterized by high levels of uncertainties and equally high stakes. As a result, they escape definitive formulations and defying absolute solutions, and only allow relative remedies (109, 110). The extent of contested values, and the capacity of affected interests to negotiate competing claims, are crucial political factors (111, 112). Laswell (113) emphasized the interdependence of knowledge contributions and value classes in a context of policy argumentation, challenging the efficacy of linear instrumental and conceptual models to explain science-policy interactions and the willingness of decision makers to utilize scientific knowledge.

These issues challenge the “conventional” approach whereby a management strategy is legitimate because it is designed by experts who resort to robust methodologies to predict and anticipate the outcomes of their actions. In such situations, legitimacy can only exist through the social consent of those likely to have a stake in the research/policy formulation or its consequences (114–116). Decision makers regularly deploy strategies to reduce the complexity of policy choice arenas, minimizing scrutiny of proposed initiatives and limiting the exploration of alternatives that correspond with stated objectives (103, 117, 118). Common strategies involve containment biases that either limit or omit the representation of contested values, or restrict knowledge and arguments to those that correspond with criteria acceptable to current political beliefs (111). Gasper and Apthorpe (119) and Cornwall (120) argue that containment biases are a function of existing power relations, constraining social values and actions, framing problems and policy solutions, and thus legitimizing certain knowledge, actions, and actors, while delegitimizing others (121, 122).

This calls for a major change in the postures and practices of health researchers and practitioners, policy makers and donors. We believe the SESH operational framework described above is relevant because it advocates for a post-normal approach (114, 117), involving an extended peer community which can provide social consent (123). With the benefit of our own experiences as practitioners supporting interventions in multi-stakeholder settings involving the types of tools and processes outlined in the Methods section (ToCs, logic models, Companion Modeling and other participatory modeling, monitoring and evaluation methods) we recognize that it takes both time and skills to facilitate SESH as an adaptive process [e.g., (99)]. As Allen et al. (124) remind us, developing a shared understanding of different viewpoints and knowledge systems is not just a matter of bringing people together. Successful collaborations require time to build a culture of trust, respect and sharing among members of the different stakeholder parties, through a combination of formal and informal interactions and relationships.

Adopting the concept of resilience to design sustainable “healthy” social-ecological systems will also imply operating at levels which are usually not handled by classical investigations in public or veterinary health (102). One critical issue in establishing resilient SES is the identification of appropriate levels where the demands on ecosystems by human societies are compatible with the quantum of services ecosystems are capable of providing (125, 126). Many of the problems encountered by societies in managing resources lie in the mismatch between the scale of management and the scale(s) of the ecological processes being managed (127). Similar problems may be expected when managing health “within/of” entire river catchments, biomes or entire agricultural systems, if the scales of the epidemiological processes and their management do not match. However, because the health and life of people, and the planet, are compelling reasons for seeking dialogue between individuals and coherence in the dimensions of socio–ecosystem sustainability (128), the transdisciplinary process prompted through SESH interventions are likely to identify the appropriate scale and stakeholders. This will nevertheless require a major shift in the policy of central governments to ensure that the devolution of the rights, responsibilities and means to manage such SESH interventions are effective through appropriate decentralized adaptive governance arrangements and operating protocols (13).

External factors and actors, operating at higher levels outside the system defined, may have key impacts on social-ecological dynamics influencing local landscapes (129). In the case of agro-ecological transitions for instance, such drivers/actors operating outside the system at national, regional or even global levels, may include reluctant dominant operators in food processing and distribution along the value-chains, associated with reduced marketing opportunities, competing agro-businesses, drug and pest-control dealers, public health and veterinary policy-makers, and extractive natural resources activities etc. For small-scale farmers, and other local stakeholders engaged in agroecological transitions, these external actors may be “out of reach,” or just not willing to take part in a participatory process, to address local issues associated with desired agricultural transitions, that may compete with their own political or economic interests. The SESH process alone will not redress such power asymmetries, and this should be clarified if and when such situations occur in order to avoid unreasonable expectations regarding the political power of the initiative and of scientific evidence (as stated in the previous paragraph). However, such resistance and blockage will be revealed and documented through the proposed SESH process, which should provide appropriate material for targeted communication, advocacy and political lobbying.

Here, we proposed an operational framework, based on the participatory, context-based and dynamic definition of Social-Ecological System Health, which promotes the active involvement of communities and stakeholders from the interlinked sectors of agriculture, public and veterinary health, and environment. Although partial, the application of our SESH operational framework in contrasted socio-cultural and professional contexts (in Boxes 1, 2) confirmed that it helps frame and facilitate fruitful transdisciplinary conversations, ultimately promoting ontological plurality (130, 131). In Vietnam and in France, it allowed us to transcend disciplines and sectors to produce shared and situated definitions of the SESH, integrating point of views, aspirations, knowledge and know-how of a variety of stakeholders. In the two case studies, using SESH as a heuristic allowed for the exploration of complex social-ecological issues associated with agricultural transitions, and the drafting of local interventions grounded in the target social-ecological systems.

Such an integrated approach, based on transdisciplinary, iterative processes, implemented to solve important issues affecting people's health (*lato sensu*), is likely to promote the emergence of adaptive governance for social–ecological resilience of landscapes, not only to current conditions and in the short-term (i.e., the SESH intervention of reference) but for decades (82). However, the implementation of our framework requires a significant paradigm shift for all stakeholders involved in the process, including donors and development agencies, acknowledging that SESH interventions address “wicked problems” which call for a post-normal scientific position to handle uncertainty, issue framing, participation, power relations and information asymmetries, politics, and attitudes toward evidence (117, 123). Adopting a participatory SESH framework will help, but it will nonetheless require a change in attitude by “experts”, donors and decision makers in order to accept that the health status (of people, animal, societies…) has to be negotiated, that local communities are co-creators of positive ways forward, and that engaging in this process, with uncertain outcomes and assessed through co-constructed indicators, is worth supporting. These paradigm shifts are necessary if we are to achieve transformations toward “healthier” development pathways, which will be one of the greatest challenges for humanity in the decades to come (82), especially in the traumatized post COVID-19 crisis context.

## Data Availability Statement

The original contributions presented in the study are included in the article, further inquiries can be directed to the corresponding author.

## Author Contributions

MG-W, AB, JW, HR, PP, RD, and PE contributed to the initial ideas and development of the rationale. MG-W, AB, JW, RD, and PE drafted the first version of the manuscript. All authors contributed to revising it critically for important intellectual content, read, and approved the final version of the manuscript.

## Conflict of Interest

The authors declare that the research was conducted in the absence of any commercial or financial relationships that could be construed as a potential conflict of interest. The handling editor HK declared a past co-authorship with several of the authors AB, SM, and RK.
